# Biliary Myeloid Sarcoma Mimicking Cholangiocarcinoma: A Diagnostic Pitfall

**DOI:** 10.7759/cureus.118025

**Published:** 2026-10-16

**Authors:** Danilo Alves, Ana Alves, Paulo Coelho, Ana Paula Borges, Paulo Donato

**Affiliations:** 1 Radiology, Hospital da Universidade de Coimbra, Unidade Local de Saúde de Coimbra, Coimbra, PRT; 2 Pathology, Hospital da Universidade de Coimbra, Unidade Local de Saúde de Coimbra, Coimbra, PRT

**Keywords:** acute myeloid leukemia, biliary myeloid sarcoma, extra-medullary myeloid sarcoma, hilar cholangiocarcinoma mimmicker, malignant obstructive jaundice

## Abstract

A 53-year-old man presented with a three-day history of jaundice and anorexia. Laboratory tests showed markedly elevated liver enzymes and bilirubin, consistent with obstructive jaundice. Computed tomography (CT) and magnetic resonance cholangiopancreatography (MRCP) demonstrated marked thickening and enhancement of the extrahepatic bile duct from the hepatic confluence to the ampulla, with intrahepatic ductal dilatation and no clear involvement of the right or left hepatic ducts.

Hilar cholangiocarcinoma (Bismuth-Corlette type II) was strongly suspected, and a multidisciplinary tumor board proposed an extended left hepatectomy with bile duct resection. The patient first underwent percutaneous biliary drainage and left portal vein embolization. Intraoperative biopsies of the right and left hepatic ducts showed no evidence of adenocarcinoma, and surgery was completed without complications.

Final pathology revealed myeloid sarcoma of the extrahepatic bile ducts, with architectural effacement by a diffuse proliferation of intermediate-sized cells with scant cytoplasm and monotonous round nuclei. The cells were diffusely positive for myeloperoxidase and multifocally positive for CD117, and negative for pancytokeratin, B-cell markers, T-cell markers, CD138, CD34, and CD163, excluding an epithelial neoplasm and lymphoma. Bone marrow involvement was subsequently identified, and the patient is awaiting chemotherapy followed by allogeneic stem cell transplantation. Many inflammatory and neoplastic biliary conditions mimic cholangiocarcinoma on imaging. Biopsy or cytology is therefore recommended before surgery.

Myeloid sarcoma, a tumor of extramedullary myeloid blasts, is equivalent to a diagnosis of acute myeloid leukemia. Primary biliary involvement is exceedingly rare and may be misdiagnosed as cholangitis or cholangiocarcinoma.

## Introduction

Cholangiocarcinoma is the second most common primary liver cancer after hepatocellular carcinoma. It is an adenocarcinoma arising from the bile duct epithelium and is generally classified as intrahepatic or extrahepatic, with each type having distinct clinical behavior and management strategies [1,2].

A wide spectrum of inflammatory and neoplastic bile duct conditions can have imaging features indistinguishable from cholangiocarcinoma [3]. A large surgical series reported that 24% of patients undergoing surgery for suspected hilar cholangiocarcinoma were ultimately found to have benign disease [4-6]. Biopsy or cytology is therefore recommended before surgical intervention [5-7]. Myeloid sarcoma (also known as granulocytic sarcoma or chloroma) is a tumor of myeloid blasts at extramedullary sites. Its detection is considered equivalent to a diagnosis of acute myeloid leukemia (AML).

Primary biliary tract involvement is exceedingly rare and can be misdiagnosed clinically as cholangitis or cholangiocarcinoma [8]. We report a case that illustrates this diagnostic pitfall.

## Case presentation

A 53-year-old man presented to the emergency department with a three-day history of jaundice and anorexia. Laboratory investigations showed marked elevation of liver enzymes and bilirubin, suggestive of obstructive jaundice.

Computed tomography (CT) demonstrated intrahepatic bile duct dilatation secondary to a markedly thickened, enhancing main biliary duct extending from the confluence to the ampullary region (Figure 1).

**Figure 1:**
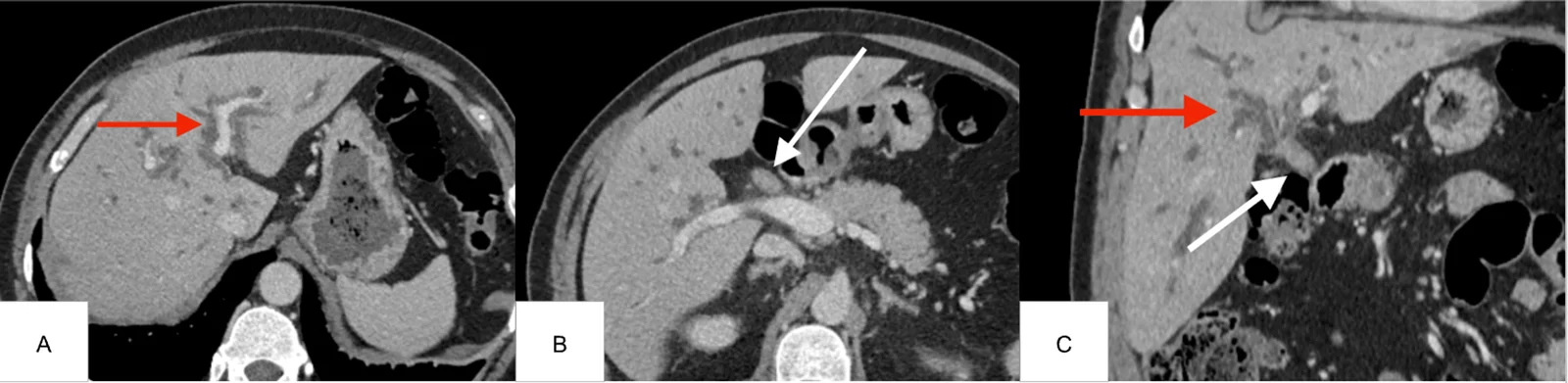
Contrast-enhanced CT findings. (A) Axial and (C) coronal contrast-enhanced CT images demonstrate marked dilatation of the intrahepatic bile ducts (red arrow). (B) Axial and (C) coronal contrast-enhanced CT images demonstrate that the cause is exuberant concentric thickening of almost the entire main biliary duct (white arrow), extending cranially to the confluence of the right and left intrahepatic ducts and distally to the ampulla of Vater.

Involvement of the main right and left intrahepatic ducts at the confluence was uncertain on CT. Magnetic resonance cholangiopancreatography (MRCP) showed marked thickening of the common hepatic duct involving the confluence of the hepatic ducts, without clear involvement of the right or left hepatic ducts (Figure 2).

**Figure 2:**
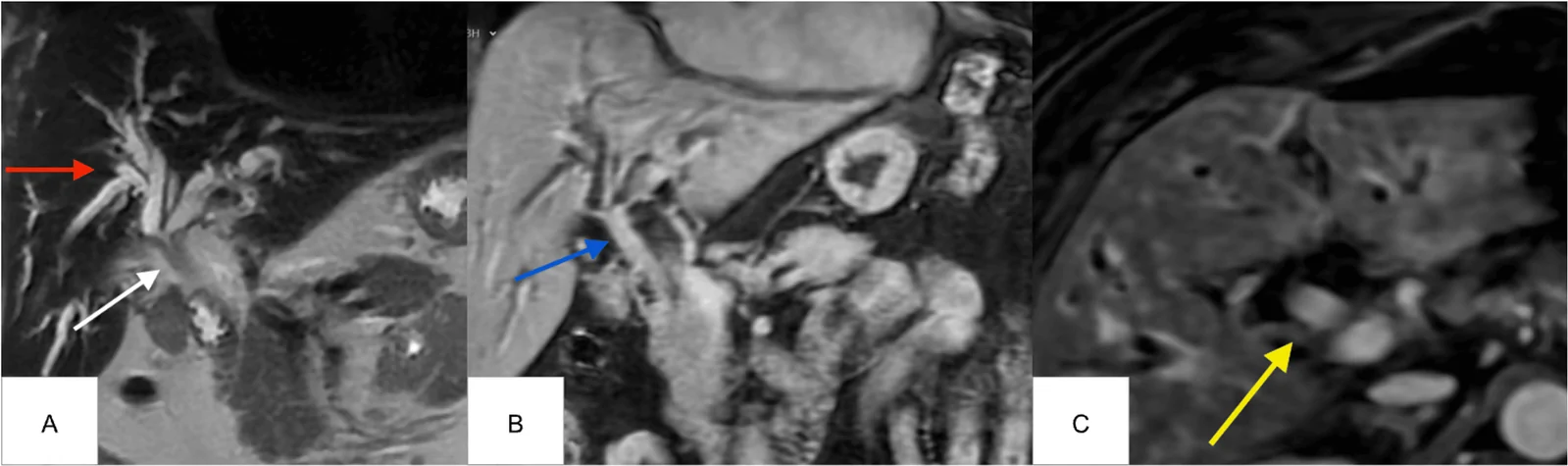
Magnetic resonance imaging findings. (A) Coronal single-shot T2-weighted image demonstrated marked dilatation of the intrahepatic bile ducts (red arrow), with significant thickening of the main biliary duct extending to the biliary confluence, without clear involvement of the right and left intrahepatic sectoral ducts. (B) Post-gadolinium coronal fat-suppressed T1-weighted image and (C) axial subtracted T1-weighted image demonstrate enhancement of the thickened main biliary duct (blue arrow in B, yellow arrow in C).

The case was discussed at a multidisciplinary tumor board. Given the high suspicion of hilar cholangiocarcinoma (Bismuth-Corlette type II), the patient was proposed for an extended left hepatectomy with resection of the main biliary duct. Before surgery, percutaneous T-tube drainage of the biliary system and left portal vein embolization were performed to induce hypertrophy of the right hepatic lobe. During surgery, biopsies of the right and left main hepatic ducts were examined intraoperatively by pathology and showed no well-differentiated adenocarcinoma. The procedure was completed without complications.

Final pathology established a diagnosis of myeloid sarcoma involving the extrahepatic bile ducts. The main bile duct, hepatic hilum, and large portal tracts showed architectural effacement by a diffuse proliferation of intermediate-sized cells with scant cytoplasm, monotonous round nuclei, dispersed chromatin, and occasional prominent nucleoli (Figure 3).

**Figure 3:**
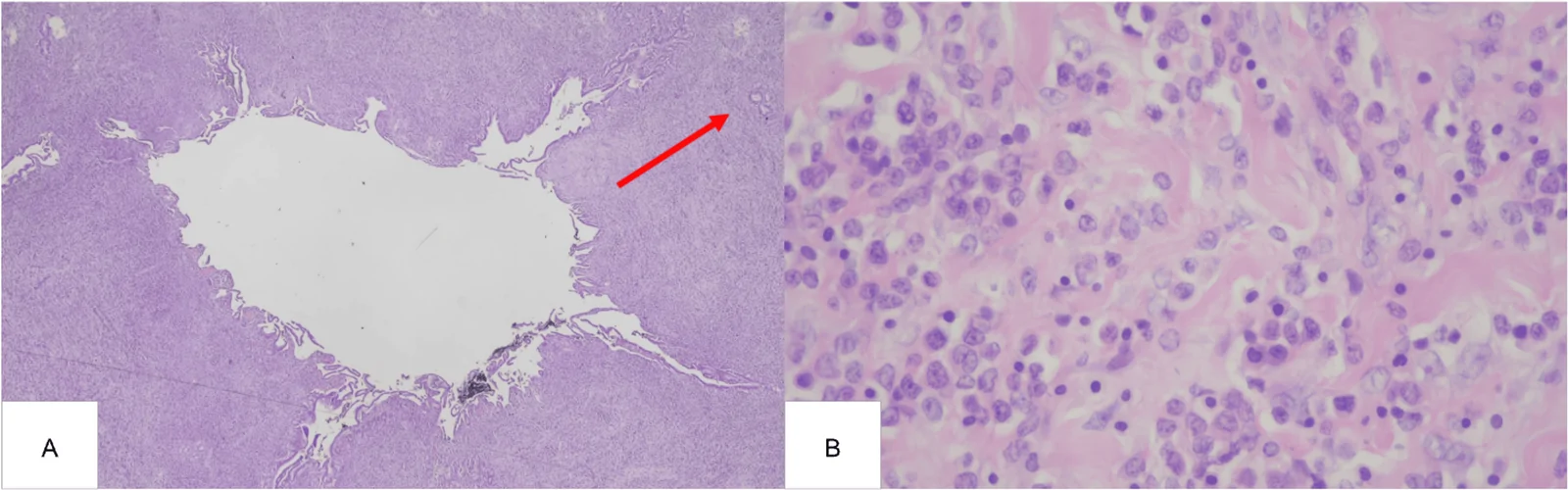
Histology of the extended left hepatectomy and main bile duct specimen. (A) Main bile duct, hematoxylin and eosin stain (20×), showing architectural effacement by a diffuse proliferation of intermediate-sized cells, with residual normal peribiliary glands (red arrow). (B) Hematoxylin and eosin stain (400×), highlighting monotonous intermediate-sized cells with scant cytoplasm and round nuclei with dispersed chromatin and occasional prominent nucleoli.

The neoplastic cells were diffusely positive for myeloperoxidase and multifocally positive for CD117. They were negative for pancytokeratins, pan-B cell markers (CD19, CD20, CD22, CD79a, PAX5), pan-T cell markers (CD2, CD3, CD5, CD7), CD138, CD34, and CD163 (Figure 4). This excluded a neoplasm of epithelial origin and B- or T-cell lymphomas.

**Figure 4:**
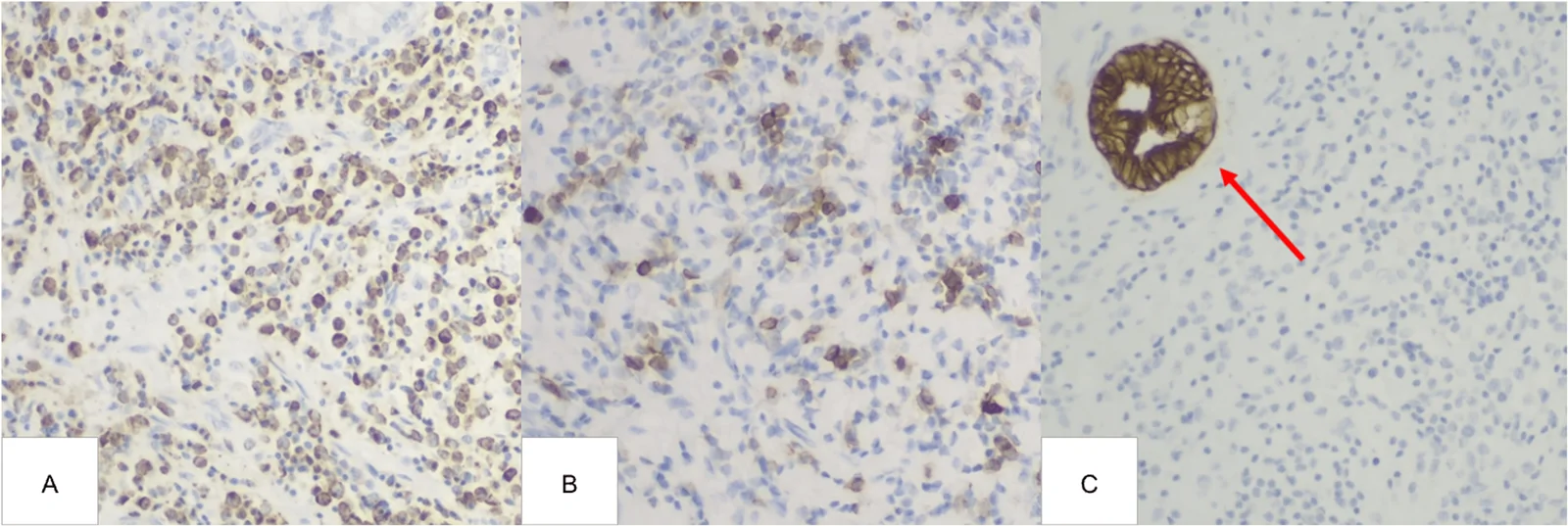
Immunohistochemistry findings. (A) Myeloperoxidase stain (200×) shows diffuse positivity in tumor cells. (B) CD117 stain (200×) shows focal positivity in tumor cells. (C) CK7 stain (200×) shows negativity in tumor cells, with an internal positive control in a peribiliary gland (red arrow).

During the preoperative work-up for a suspected hilar cholangiocarcinoma, a peripheral blood sample revealed 3.5% of cells with blast morphology. Immunophenotyping was positive for CD117 and negative for CD34, HLA-DR, CD19, CD79a, and CD3. Given the suspicion of AML, the patient was referred to the hematology team, and a bone marrow aspirate and biopsy were scheduled to establish the diagnosis.

Bone marrow involvement was subsequently confirmed, establishing the diagnosis of AML. Until the final pathology of the surgical specimen became available, the biliary lesion and the leukemia were regarded as two independent diseases occurring concurrently. Only after surgery was it recognized that the presumed Klatskin tumor was in fact a myeloid sarcoma, an extramedullary manifestation of the patient’s AML. The patient was recommended chemotherapy followed by allogeneic hematopoietic stem cell transplantation and is currently undergoing treatment.

## Discussion

This case shows that imaging alone is insufficient to diagnose hilar cholangiocarcinoma. Several benign and malignant conditions can closely mimic it on CT and MRCP, and up to one quarter of patients operated on for suspected hilar cholangiocarcinoma have non-cholangiocarcinoma pathology. Preoperative tissue diagnosis should therefore be obtained whenever feasible [3,4].

Although rare, extramedullary myeloid sarcoma can involve the extrahepatic bile ducts and present clinically and radiologically like a Klatskin tumor. It should be considered in the differential diagnosis, especially when imaging findings are atypical or intraoperative biopsies are negative, as in this patient [5-7].

Histopathology and immunohistochemistry are critical for definitive diagnosis. Myeloperoxidase and CD117 positivity, together with exclusion of epithelial markers, are essential to distinguish myeloid sarcoma from cholangiocarcinoma. The distinction changes management dramatically, from major hepatobiliary surgery to systemic chemotherapy and stem cell transplantation [8].

## Conclusions

Myeloid sarcoma of the extrahepatic bile ducts is an exceedingly rare entity that can closely mimic hilar cholangiocarcinoma, both clinically and on CT and MRCP. In this patient, imaging findings and the clinical presentation led to a high suspicion of Bismuth-Corlette type II hilar cholangiocarcinoma and to extended hepatic surgery, while the correct diagnosis was established only on final histopathology and immunohistochemistry. Preoperative tissue diagnosis should be obtained whenever feasible in suspected hilar cholangiocarcinoma, and myeloid sarcoma should be considered in the differential diagnosis when imaging is atypical or intraoperative biopsies are negative. Because this diagnosis is equivalent to AML and shifts treatment from major hepatobiliary surgery to systemic chemotherapy and stem cell transplantation, accurate and timely recognition has a direct impact on patient management.
